# Thermoplastic Laminated Composites Applied to Impact Resistant Protective Gear: Structural Design and Development

**DOI:** 10.3390/polym15020292

**Published:** 2023-01-06

**Authors:** Yan Yu Lin, Mei-Chen Lin, Ching-Wen Lou, Yueh-Sheng Chen, Jia-Horng Lin

**Affiliations:** 1 Laboratory of Fiber Application and Manufacturing, Advanced Medical Care and Protection Technology Research Center, Department of Fiber and Composite Materials, Feng Chia University, Taichung 407102, Taiwan; 2 Department of Biomedical Engineering, College of Biomedical Engineering, China Medical University, Taichung 404333, Taiwan; 3 Fujian Key Laboratory of Novel Functional Fibers and Materials, Minjiang University, Fuzhou 350108, China; 4 Department of Bioinformatics and Medical Engineering, Asia University, Taichung 413305, Taiwan; 5 Department of Medical Research, China Medical University Hospital, China Medical University, Taichung 404327, Taiwan; 6 Innovation Platform of Intelligent and Energy-Saving Textiles, School of Textile Science and Engineering, Tiangong University, Tianjin 300387, China; 7 Advanced Medical Care and Protection Technology Research Center, College of Textile and Clothing, Qingdao University, Qingdao 266071, China; 8 School of Chinese Medicine, China Medical University, Taichung 404333, Taiwan; 9 College of Material and Chemical Engineering, Minjiang University, Fuzhou 350108, China

**Keywords:** Kevlar, TPU, laminated composites, flexibility

## Abstract

Laminated composites have been commonly applied to all fields. When made into laminated composites, Kevlar woven fabrics are able to provide the required functions. In this study, two types of TPU are incorporated to improve the intralayer features of Kevlar/TPU laminated composites. Hence, the Kevlar/TPU laminated composites consist of firmly bonded laminates while retaining flexibility of the fabrics. Being the interlayer of the laminated composites, the TPU layer provides adhesion while strengthening the tensile property, dynamic puncture resistance, and buffer strength of Kevlar/TPU laminated composites. The test results indicate that with a blending ratio of two types of TRU being 85/15 wt%, the Kevlar/TPU laminated composites exhibit a tensile strength of 18.08 MPa. When the stacking thickness is 1 mm, the tensile strength is improved to 357.73 N with the buffering strength reaching 4224.40 N. Notably, with a thickness being 1.2 mm, the laminated composites demonstrate a dynamic resistance being 672.15 N. In the meanwhile, functional Kevlar fabrics are allowed to keep the fiber morphology owing to the protection of TPU composite films. Considering the composition of protective gear, Kevlar/TPU laminated composites possess a powerful potential and are worthwhile exploring.

## 1. Introduction

There are increasing demands on composites in terms of good mechanical properties and a light weight because the development in composites has been flourishing. In the aviation, protection, and construction fields, composites can be effectively reinforced via the employment of lamination techniques and hot-pressing process, as well as a combination of multiple layers of materials, producing composites with the desired mechanical features and functions. Composed of fibers and polymers, the hot-pressing laminated composites take advantage of the fact that fibers can be combined with polymers, thereby achieving lamination bonding and external force dispersion. Moreover, polymer matrices do not alter their intrinsic attributes after they are embedded with fibers [1,2,3,4,5]. 

There are many studies applying lamination and hot pressing to form the basic structure of composites whereas some studies address potential problems that the laminated composites may encounter. Attahu et al. combined carbon fibers and aramid fibers to form composite nonwoven fabrics, and then the composites and epoxy resin were laminated with a titanate coupling agent as the intralayer adhesive. They reported that the amount of the adhesive had a negative influence on the bending performance of the resulting laminated composites [6]. Sitohang et al. proposed thermoplastic laminated composites that exhibited a shear force in sporadic areas because of the misaligned or twisted intralayer fibers, which eventually caused the failure of composites [7,8]. In addition, the properties and failure mode of the hot-pressing laminated composites were dependent on the expansion of intralayer fibers [9], the twisting of lamination planks [10], or the delamination of laminated composites [11]. In particular, delamination is one of the major factors that causes damage to composites. As hot-pressing laminated composites are usually composed of two or more than two layers of different materials that are firmly adhered, the occurrence of delamination is ascribed to adhesion failure. Possible factors include intralayer damage, geometric details, manufacturing flaws, or out-of-plane loading, all of which may be spread in the interfaces among the laminated layers. That fibers and matrices are well bonded and bridged can be an effective measure to address the delamination problem, strengthening the bonding force over the interfaces of laminated composites [12,13,14,15,16]. 

The use of high-modulus fibers expands the development domains of composites. A combination of laminated structures strengthens the mechanical properties and energy absorption capacity of composites [17]; however, the pullout or breakage of fibers, delamination, and cracks in the matrices also cause the failure of the materials [18,19,20,21]. In light of the background of the study and related literature, multiple structures have a positive influence over the rigidness of laminated composites whereas intralayer failure mechanisms render the composites with failure. Important manufacturing parameters for laminated composites involve the material type, multiple-stage process, and construction design. According to our previous studies, high-modulus fabrics and TPU films are combined, thereby examining the effects of the density structure. It is proven that the composite planks exhibit excellent resilience in the peeling test. The test results suggest that a firm adhesion prevents the composites from peeling off or delamination when an external force is exerted. 

With high modulus Kevlar fibers contributing to the composites with mechanical strength, it is emphasized to strengthen the bonding force of the Kevlar/TPU laminated composites in this study. Two different TPU types are blended as the adhesive, thereby evaluating the effect of adhesive over the delamination phenomenon. Blending TPUs can reduce the interface area among different materials, and two similar TPU types are processed in a manageable temperature range. TPU has a resilience that provides the composites with bending properties, and simultaneously serving as an adhesive, TPU also strengthens the Kevlar/TPU laminated composites via the fiber-matrix bridge effect. The composites can be used as an interlayer that is incorporated with protective gear, including protective clothing, protective hats, and protective gloves. 

## 2. Materials and Methods

### 2.1. Materials

Two types of thermoplastic polyurethane (Headway Polyurethane, Hsinchu, Taiwan) are used. One is HE-3285ALE, which has a rigidity of 87 A, a melting point of 140 °C, a modulus of 55 kg/cm^2^, an MI of 7.03 g/10 min, and a melting viscosity of 2818 POISE. The other TPU is eco-friendly MTPU (HE-3580AU) that is used as an adhesive with a rigidity of 76–84 A, a melting point of 100 °C, a modulus of 40 kg/cm^2^, MI of 7.25 g/10 min, and a melting viscosity of 3000 POISE. TPU and MTPU are basically composed of the same elements but at different ratios that are ascribed to different physical properties, such as crystallinity, melting point, and softening point. Besides, MTPU is to replace the glue as an adhesive and therefore exhibits a lower melting point, e.g., 70~110 °C. MTPU can be melted at lower temperatures to attain the adhesion function. Kevlar woven fabrics (DuPont, Miaoli, Taiwan) have a plain weave, composed of a yarn fineness being 1500 filament/strand, a warp/weft density being 15 ends (picks)/inch, and a thickness being 0.35 mm.

### 2.2. Preparation Process for TPU Composite Films and Kevlar/TPU Laminated Composites

**Figure 1:**
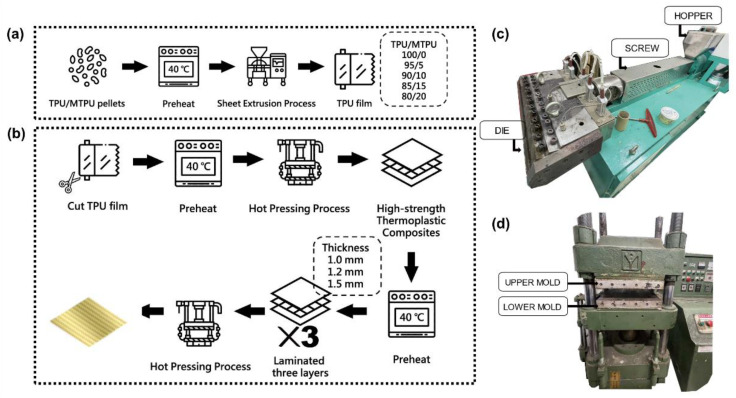
Process diagrams of (**a**) TPU planks and (**b**) Kevlar/TPU laminated composites. Images of (**c**) film extrusion assembly and (**d**) hot pressing assembly.

**Figure 2:**
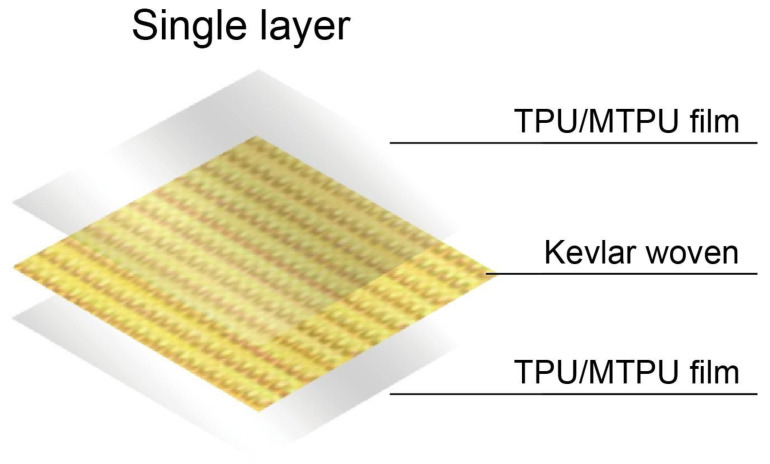
Illustrative diagram of the structure of a single TPU film.

TPU and MTPU are thermally baked at 40 °C for overnight, thereby removing the residual moisture in advance. A single-screw sheet extrusion process is employed to produce TPU films as Figure 1a–c shows the assembly. The TPU/MTPU ratios are 100/0, 95/5, 90/10, 85/15, and 80/20 wt%, and the resulting TPU films are tested and evaluated in order to acquire the optimal parameter. Next, the hot-pressing process is conducted to combine Kevlar woven fabrics, thereby producing Kevlar/TPU laminated composites. The hot pressing time and pressure are ten minutes and 50 kg/cm^2^, respectively. The upper and lower mold is 160 °C. Figure 1d shows the hot pressing equipment that is heated to 160 °C in advance while Figure 2 shows the diagram of the hot pressing process. To begin with, a sample is mounted in the mold set with a specified thickness (i.e., 1.0, 1.2, and 1.5 mm), and the mold is then placed in the hot pressing assembly. In order to evenly heat the materials for a better melting state, the upper and lower molds are sealed for the first two minutes. Next, the lower mold is moved back and forth three to four times, ensuring the sample is firmly adhered, and the lower mold is moved upward to heat the sample along with the upper mold the last time for six minutes. At last, the iron plates are removed for cooling, after which the Kevlar/TPU laminated composites are evaluated for morphology and applications. 

### 2.3. Measurements

A stereo microscope is employed to observe the surface of samples while a scanning electron microscope (SEM) is employed to observe the micro-structure of samples. The thermal behavior of samples is observed as follows. Sample weighing 9~10 mg is sealed in an aluminum plate and measured using a DSC (Q20, TA Instruments, New Castle, USA). The DSC measurement is conducted in a temperature range that is increased from 40 °C to 200 °C by increments of 10 °C/min, during which, samples are observed for the melting status and crystallization morphology. The tensile strength of samples is measured at a test rate of 5 mm/min using the Instron 5566 Universal Tester (Norwood, USA) as specified in ASTM D638-10. Samples are dumbbell-shaped (Type IV). A drop weight impact tester (Changfata Industrial Company, Taichung, Taiwan) is used in both the dynamic puncture resistance and buffer strength tests. As for the dynamic puncture resistance test, the impact mass of the impactor is 20 kg, and the impactor is released from a specified height of 450 mm. Samples have a size of 100 mm × 100 mm. The transient maximal force that penetrates the sample is recorded as the anti-puncture value. In addition, as specified in ASTM D1596, the drop weight impact resistance measurement is conducted to evaluate the buffer strength of samples (100 mm × 100 mm). The impactor weighing 10 kg is released from a specified height of 200 mm in order to strike the sample, during which the sensor linked with a computer detects the maximal absorption capacity for buffer strength evaluation. For all measurements, ten samples for each specification are used. 

## 3. Results and Discussion 

### 3.1. Tensile Performance of TPU Composite Films

**Figure 3:**
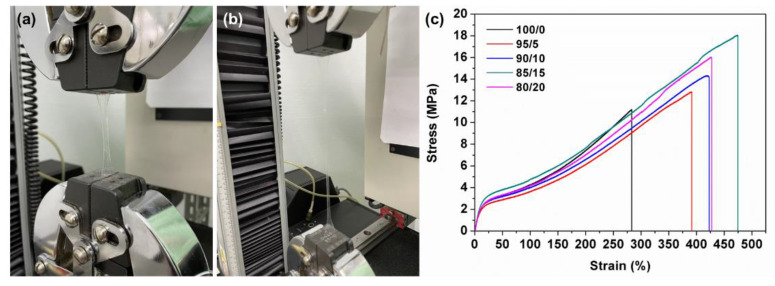
(**a**,**b**) Tensile test process and (**c**) tensile properties of TPU planks.

Composites consist of two or more than two materials with different characteristics, and the materials are bonded via physical, chemical, physical-chemical, or mechanical mechanisms. In this study, two types of TPU are combined to form TPU composite films, thereby bettering the tensile performance of the composite films as well as strengthening the adhesion level among laminates [22,23,24]. The films can be adjusted according to the thickness and the resulting films exhibit a more even morphology than other films made by molds of various thicknesses via hot pressing. Figure 3 shows the tensile stress-strain curves of TPU composite films, and all of the TPU composite films demonstrate good tensile features regardless of the TPU/MTPU blending ratios. In addition, the greater the MTPU content, the higher the tensile stress and strain of TPU composite films. However, with a TPU/MTPU ratio being 80/20, the tensile stress and strain start to decline. The TPU composite films show a raise in the tensile strain because MTPU (the adhesive type TPU) is well mixed with TPU and contributes greater tensile properties. Nevertheless, when the content of MTPU exceeds the limit, there is subsequently a smaller space for TPU chain linking, which in turn has a negative influence over the tensile stress and strain. 

### 3.2. DSC Observation of TPU Composite Films

**Figure 4:**
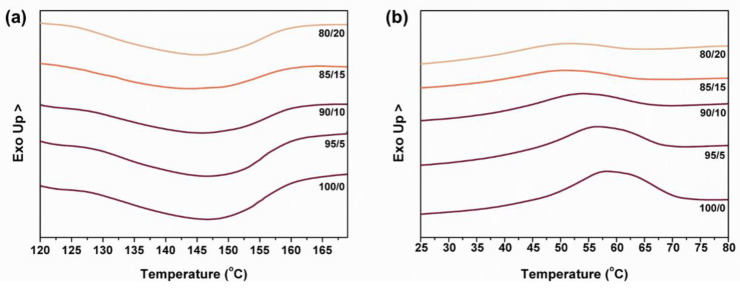
DSC curves of (**a**) melting and (**b**) crystallization of TPU composite films.

Figure 4 shows the DSC curves of TPU composite films. To testify to the thermal behaviors of TPU composite films during the melting process, the melting and crystallization characteristic peaks are observed. Figure 4a shows that with the incorporation of MTPU, the melting characteristic peak of TPU composite films has a lower and wider peak. Figure 4b shows the comparable melting and crystallization characteristic peaks, and in the meanwhile, the crystallization degree shows a declining trend after MTPU and TPU are blended. TPU has a structure consisting of hard and soft segments that are alternatively aligned. Hard segments provide crystallization sites while soft segments help with the expansion of the TPU composite films [25,26]. Specifically, the soft segments could be entangled with each other, which strengthens the blending level of TPU and MTPU subsequently. In this study, the aim is to explore the application feasibility of combining TPU composite films and fabrics. Hence, MTPU is used to bond laminates as an adhesive and its resilient attribute improves the protection of the fabric layers.

### 3.3. Composition and Morphology of Kevlar/TPU Laminated Composites

**Figure 5:**
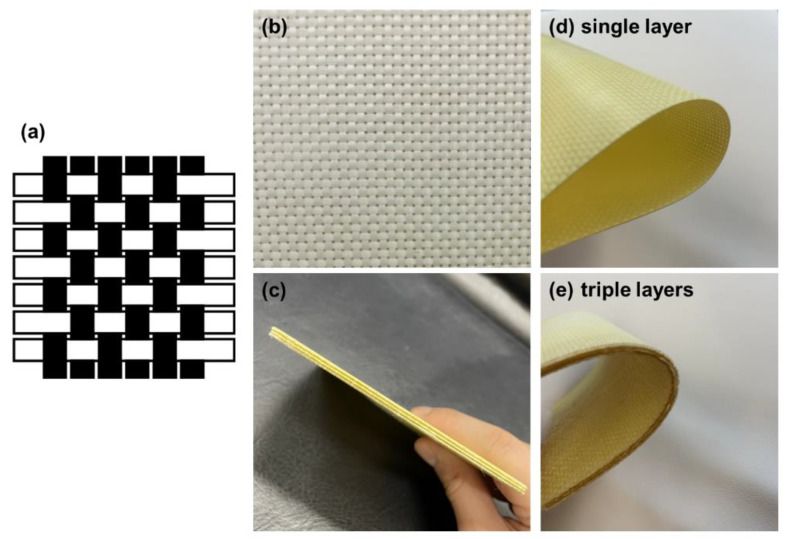
Morphology of Kevlar/TPU laminated composites. (**a**) woven structure, (**b**) plane and (**c**) cross-sectional photos of the sample, (**d**) single-layer fabric, (**e**) three-layer fabric.

Figure 5 demonstrates the surface observation of Kevlar/TPU laminated composites where Kevlar fabrics have a woven structure. The good mechanical properties of hot-pressing laminated composites are attributed to the interlacing warp and weft yarns. The risers mean the point where the warp and weft yarns are crossed, whether it is with the warp or the weft yarns being on top of the opposite yarn. Subsequently, TPU infiltrates along the path offered by the risers. Comprising the composites, the constituent fibers are ubiquitous throughout the whole structure, and as such attain isotropic reinforcement. Namely, Kevlar/TPU laminated composites have a multi-layered structure and the alternatively aligned TPU films serve as adhesive layers. As a result, the laminated composites are well-bonded while preserving the softness of fabrics and films concurrently. Similarly, the three-layered Kevlar/TPU laminated composites exhibit the softness attribute. The combination of TPU composite films and Kevlar woven fabrics successfully retains the mechanical properties of Kevlar woven fabrics. Despite the conduction of the sheet extrusion process and hot-pressing lamination, TPU composite films still keep the flexible attribute. 

### 3.4. Tensile Properties of Kevlar/TPU Laminated Composites

**Figure 6:**
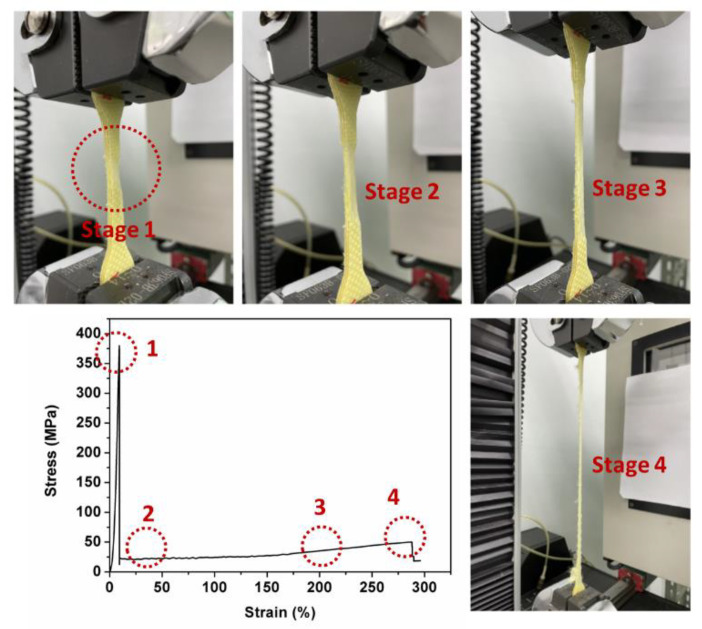
Tensile test process of Kevlar/TPU laminated composites.

**Figure 7:**
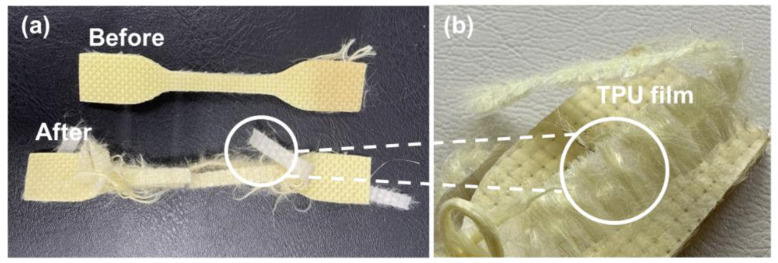
(**a**) Tensile test results and its (**b**) microstructure of Kevlar/TPU laminated composites.

Figure 6 and Figure 7 demonstrate the tensile test results of Kevlar/TPU laminated composites. As shown in Figure 6, Kevlar/TPU laminated composites are affixed by a pair of clamps, and then are stretched by the clamps in opposite directions until samples are broken. During the tensile test, Kevlar woven fabrics are the subject that is first broken. In the meanwhile, a small proportion of fibers remain embedded in the TPU composite films without being exposed during and after the tensile test. Figure 7 shows the results of tensile stress and strain tests, the broken samples demonstrate a trivial amount of Kevlar fibers embedded in TPU composite films, thereby limiting the expansion of TPU composite films. This result is in conformity with the results of the tensile strain test. To sum up, the tensile strain of Kevlar/TPU laminated composites is only 295.94%, which is decreased by 60.32%. Kevlar/TPU laminated composites exhibit two-stage breakage, which suggests that Kevlar woven fabrics and TPU composite films are separately damaged in order. By contrast, the Kevlar/TPU laminated composites exhibit greater tensile stress than that of TPU composite films (85/15 wt%), which is 49.96 Mpa and 18.08 Mpa, respectively. As Kevlar/TPU laminated composites are composed of two different structures with corresponding materials, delamination occurs inevitably. Two constituent materials confine each other while strengthening the whole structure of Kevlar/TPU laminated composites. Subsequently, the embedded Kevlar fibers exert reinforcement over the residual TPU composite films. Although Kevlar/TPU laminated composites have multiple layers, they demonstrate two maximal tensile strengths exclusively, indicating that fabrics and films are bonded in a one-piece state.

### 3.5. Effects of Thickness on Tensile Properties of Kevlar/TPU Laminated Composites 

**Figure 8:**
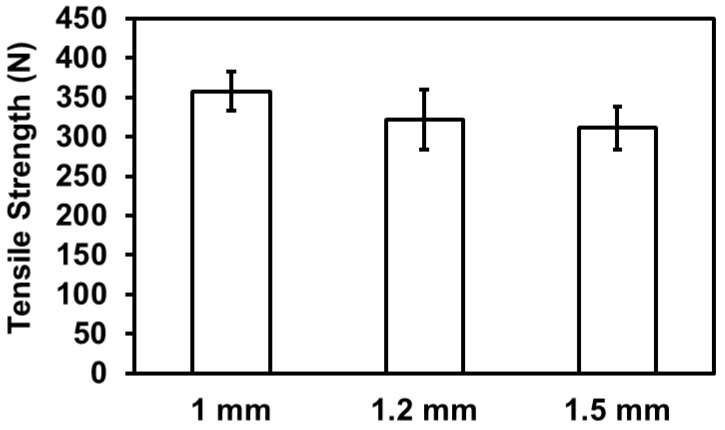
Tensile strength of Kevlar/TPU laminated composites as related to the thickness.

Figure 8 shows the tensile properties of Kevlar/TPU laminated composites as related to the thickness of samples, including 1 mm, 1.2 mm, and 1.5 mm. The tensile strength of samples is inversely proportional to the thickness of samples, decreasing from 357.73 N to 311.18 N. There is a significant association between the thickness of samples and the density of the lamination [27]. It is apparent that TPU layers show a greater compression capacity while strengthening the intralayer bonding force remarkably. The Kevlar/TPU laminated composite has a thickness of 0.5 mm, and three layers account for an original thickness of 1.5 mm. By contrast, the three-layered Kevlar/TPU laminated composites are hot pressed into a thickness of 1 mm, which suggests that TPU is melted to infiltrate Kevlar woven fabrics, achieving a more compact intra-layer structure. Therefore, this specified group exhibits maximal tensile strength. However, when hot pressed in a mold with a thickness of 1.2 or 1.5 mm, there is less restriction in the space for the laminated composites, especially the 1.5-mm group. The brief melting-cooling intervals of the hot pressing process hamper TPU from infiltrating the composites, which in turn compromises the tensile performance of Kevlar/TPU laminated composites. 

### 3.6. Dynamic Puncture Resistance and Impact Resistance of Kevlar/TPU Laminated Composites

**Figure 9:**
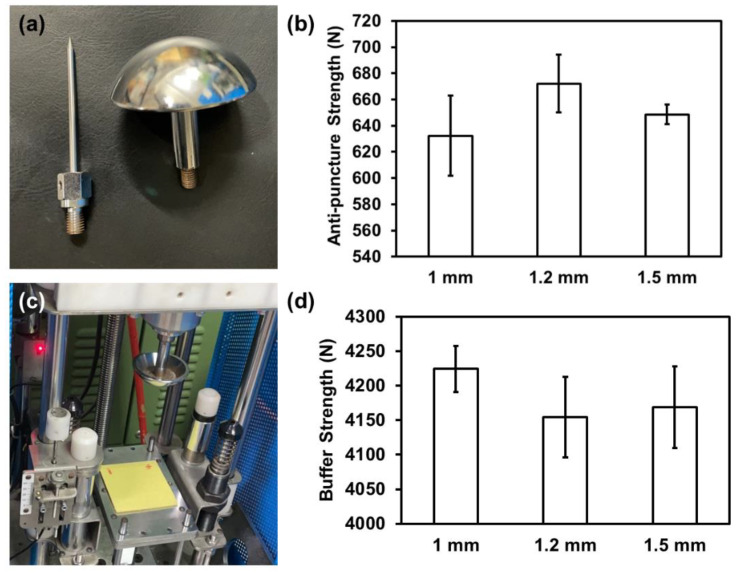
Anti-puncture and buffer strengths of Kevlar/TPU laminated composites as related to the thickness. (**a**) Test mold, (**b**) puncture test result, (**c**) impact device, (**d**) buffer test result.

Figure 9 shows the dynamic anti-puncture strength and buffer strength of Kevlar/TPU laminated composites. The thickness of samples is specified in both tests. Figure 9a shows two impactors for the corresponding tests. The needle-type impactor is for the dynamic anti-puncture test while the rounded impactor is used in the buffer strength test. Figure 9b shows the anti-puncture strength of Kevlar/TPU laminated composites as related to the thickness, and the anti-puncture strength first increases and then decreases. With the thickness increasing from 1 mm, 1.2 mm to 1.5 mm in order, the tensile strength declines to 632.30 N, 672.14 N, and then 648.52 N correspondingly. By contrast, the buffer strength of Kevlar/TPU laminated composites exhibits different trends. Figure 9c demonstrates the buffer strength test assembly. Right after the rounded impactor strikes the sample, the sensor transmits the residual buffer strength, which is then recorded to evaluate how the sample buffers the impact force. Finally, Figure 9d shows the buffer strength of Kevlar/TPU laminated composites as related to the thickness of samples. The test results indicate that a rise in the thickness adversely affects the buffer level of Kevlar/TPU laminated composites, which decreases from 4224.39 N to 4168.89 N. Two different trends in the two different tests (puncture resistance and impact resistance tests) are attributed to the damage mechanisms. With a needle-like impactor, the puncture resistance of composites is dependent on the overall structure. As for Kevlar/TPU laminated composites, the 1.2-mm (thick) group has a lower intralayer density than the 1-mm group, and therefore the multiple Kevlar fabrics may block the needle-like impactor. Nonetheless, the 1.5-mm group with a lower density of adhesion layer may show the displacement of woven fabric structure, which is responsible for a decreasing trend in the puncture resistance of the Kevlar/TPU laminated composites of a 1.5-mm thickness [28,29,30]. 

As for the impact resistance performance, it is the TPU layer that provides the dominate cushion effect. The 1-mm group is composed of a more tightly formed TPU layer that comes through the three-layered Kevlar/TPU laminated composites, and the TPU layer thus contributes higher impact resistance (buffer effect). By contrast, the 1.2- and 1.5-mm groups consist of a comparatively lower structural density, so the corresponding three-layered Kevlar/TPU laminated composites demonstrate lower impact energy dissipation in the impact resistance test [31,32,33].

**Figure 10:**
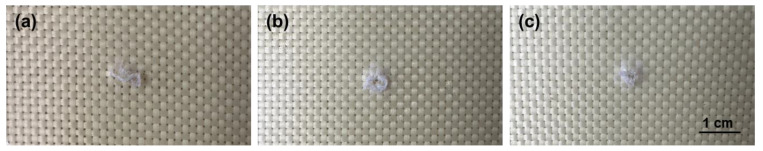
Images of punctured Kevlar/TPU laminated composites as related to the thickness of (**a**) 1, (**b**) 1.2, and (**c**) 1.5 mm.

**Figure 11:**
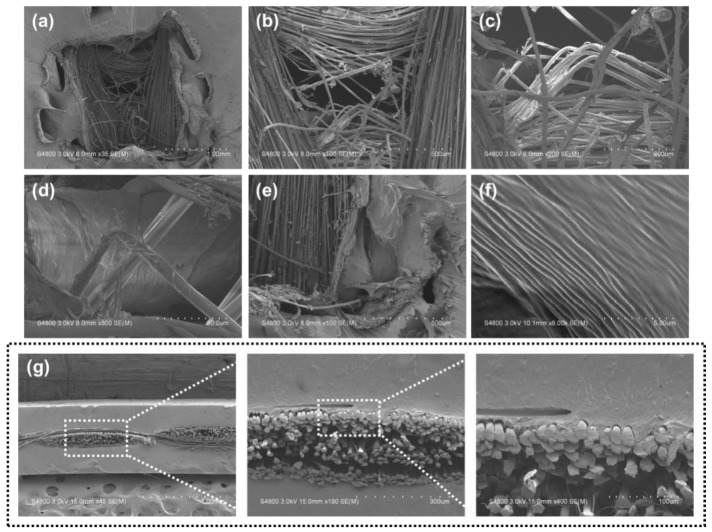
(**a**) SEM photos of Kevlar/TPU laminated composites after the puncture, (**b**–**d**) the position after the fiber is damaged, (**e**,**f**) the position after the sheet is damaged, (**g**) section photo of laminated composites.

Figure 10 and Figure 11 show photos of samples of Kevlar/TPU laminated composites after the puncture. The results show that under a high-speed impact, three different composite laminates are damaged on the surface, forming a sharp needle residue hole, and the SEM images show the microscopic damage position. The Kevlar fiber entanglement level is associated with the infiltration level of TPU. Figure 10a shows that the 1-mm group has the least number of fibers appearing in the surface, which is owing to a compact material structure. However, Figure 10b,c shows that 1.2- and 1.5-mm groups demonstrate higher puncture resistance with a small amount of fiber embedded in TPU. The fibers are thus evenly formed into a circular state over the surface due to the needle-like impactor, and the fiber counts are lower than that in Figure 10a.

Figure 11a shows that both the thin TPU composite film and the Kevlar woven fabric are damaged at the punctured position, while Figure 11b–d shows that when the fabric is punctured, the needle penetrates against the fabric, which generates strong friction and causes the fibers to become finer until they are broken. The puncture resistance can be confirmed by the fiber surface showing much hairiness in the image [34]. Figure 11e,f shows that the thin film is squeezed after being punctured and ruptured layer by layer around the damaged position. The extruded thin film is also damaged due to the slippage and fracture of the fibers at the bottom layer. The photographs of the fibers and sheets are shown in Figure 11g, the fibers are embedded in the films and bonded to each other. Limiting the thickness of the Kevlar/TPU laminated composites by the hot-pressing method can improve the bonding effect between layers and achieve a good reinforcing effect in the mechanical property tests [35,36]. The proposed Kevlar/TPU laminated composites only allow a lower content of the TPU blend infiltrating Kevlar fibers. The interweaving of woven fabrics strengthens the intralyer adhesive mechansim, and the interweaving of warp yarns and weft yarns generate rugged surface. The convex parts of woven fabrics are combined with TPU, which simultaneously drawing the concave parts of the woven fabrics. According to the peeling characteristic that is previously discussed, this manner of combination retains the flexible attribute of the laminated composites while protecting and strengthening the fiber structure. 

## 4. Conclusions

In this study, Kevlar/TPU laminated composites were successfully prepared via the sheet extrusion process and hot-pressing process. For the starter, TPU composite films were formed with a greatly even structure because of the employment of the sheet extrusion process. In particular, with a TPU/MTPU blending ratio of 85/15 wt%, TPU composite films attained the optimal tensile properties. Next, the TPU composite films were melted to infiltrate the Kevlar woven fabrics during the hot-pressing process, which bonded the composites well. The mechanical properties of TPU composite films were highly improved with the incorporation of Kevlar woven fabrics. The 1-mm-thick group exhibited a maximal tensile strength as high as 357.73 N. According to the dynamic anti-puncture test and buffer strength test, the presence of TPU was proven to firmly adhere to different laminates. Moreover, TPU composite films demonstrated different extents in the anti-puncture strength and buffer strength performances when the intralayer density and failure mode were changed. In conclusion, the proposed Kevlar/TPU laminated composites can be used as an interlayer of the protective gear and embody powerful flexibility, qualifying Kevlar/TPU laminated composites as a reinforcement material. 

## Data Availability

Not applicable.

## References

[1] Liu Z., Wang H., Yang L., Du J. (2022). Research on mechanical properties and durability of flax/glass fiber bio-hybrid FRP composites laminates. Compos. Struct..

[2] Cho S.-Y., Yu H., Choi J., Kang H., Park S., Jang J.-S., Hong H.-J., Kim I.-D., Lee S.-K., Jeong H.S. (2019). Continuous Meter-Scale Synthesis of Weavable Tunicate Cellulose/Carbon Nanotube Fibers for High-Performance Wearable Sensors. ACS Nano.

[3] Sánchez-Adsuar M.S., Linares-Solano A., Cazorla-Amorós D., Ibarra-Rueda L. (2003). Influence of the nature and the content of carbon fiber on properties of thermoplastic polyurethane-carbon fiber composites. J. Appl. Polym. Sci..

[4] Yin X., Wang L., Li S., He G., Yang Z., Feng Y., Qu J. (2016). Preparation and characterization of carbon fiber/polylactic acid/thermoplastic polyurethane (CF/PLA/TPU) composites prepared by a vane mixer. J. Polym. Eng..

[5] Radzi A.M., Sapuan S.M., Jawaid M., Mansor M.R. (2017). Influence of fibre contents on mechanical and thermal properties of roselle fibre reinforced polyurethane composites. Fibers Polym..

[6] Attahu C.Y., Thein C.K., Wong K.H., Yang J. (2022). Enhanced damping and stiffness trade-off of composite laminates interleaved with recycled carbon fiber and short virgin aramid fiber non-woven mats. Compos. Struct..

[7] Sitohang R., Grouve W., Warnet L., Wijskamp S., Akkerman R. (2022). The relation between in-plane fiber waviness severity and first ply failure in thermoplastic composite laminates. Compos. Struct..

[8] Kutty S.K.N., Nando G.B. (1991). Short kevlar fiber–thermoplastic polyurethane composite. J. Appl. Polym. Sci..

[9] Zhao C., Ren R., Zhong J., Goh K.L., Zhang K., Zhang Z., Le G. (2022). Intralaminar crack propagation of glass fiber reinforced composite laminate. Structures.

[10] Anuse V.S., Shankar K., Velmurugan R., Ha S.K. (2022). Compression-After-Impact analysis of carbon fiber reinforced composite laminate with different ply orientation sequences. Int. J. Impact Eng..

[11] O’Donnell J., Chalivendra V. (2021). Multi-functional glass/carbon fibers hybrid inter/intra laminated composites. Compos. Part C Open Access.

[12] Trabal G.G., Bak B.L.V., Chen B., Jensen S.M., Lindgaard E. (2022). Delamination toughening of composite laminates using weakening or toughening interlaminar patches to initiate multiple delaminations: A numerical study. Eng. Fract. Mech..

[13] Russo P., Langella A., Papa I., Simeoli G., Lopresto V. (2016). Low-velocity Impact and Flexural Properties of Thermoplastic Polyurethane/Woven Glass Fabric Composite Laminates. Procedia Eng..

[14] Kutty S.K., Nando G.B. (1993). Self adhesion of short Kevlar fibre-thermoplastic polyurethane composite. J. Adhes. Sci. Technol..

[15] Boyd S.E., Bogetti T.A., Staniszewski J.M., Lawrence B.D., Walter M.S. (2018). Enhanced delamination resistance of thick-section glass-epoxy composite laminates using compliant thermoplastic polyurethane interlayers. Compos. Struct..

[16] Vajrasthira C., Amornsakchai T., Bualek-Limcharoen S. (2002). Fiber-matrix interactions in aramid-short-fiber-reinforced thermoplastic polyurethane composites. J. Appl. Polym. Sci..

[17] Liu H., Falzon B.G., Dear J.P. (2019). An experimental and numerical study on the crush behaviour of hybrid unidirectional/woven carbon-fibre reinforced composite laminates. Int. J. Mech. Sci..

[18] Liu H., Zhou J., Kong X., Li S. (2022). Fracture behaviour of fibre-reinforced composite materials subjected to shear loading: An experimental and numerical study. Int. J. Lightweight Mater. Manuf..

[19] Chen Y., Hou S., Fu K., Han X., Ye L. (2017). Low-velocity impact response of composite sandwich structures: Modelling and experiment. Compos. Struct..

[20] Chen Y., Ye L., Escobedo-Diaz J.P., Zhang Y.-X., Fu K. (2019). Quasi-static and dynamic progressive crushing of CF/EP composite sandwich panels under in-plane localised compressive loads. Compos. Struct..

[21] Chen Y., Ye L., Escobedo-Diaz J.P., Zhang Y.X. (2020). Effect of initiator geometry on energy absorption of CFRP tubes under dynamic crushing. Int. J. Crashworthiness.

[22] Azammi A.M.N., Sapuan S.M., Ishak M.R., Sultan M.T.H. (2018). Mechanical and Thermal Properties of Kenaf Reinforced Thermoplastic Polyurethane (TPU)-Natural Rubber (NR) Composites. Fibers Polym..

[23] Ren L., Ren Y., Zhang Y., Orzechowski K., Kułacz K., Pocheć M., Bai S.-L. (2020). Graphite films/carbon fiber fabric/ polyurethane composites with ultrahigh in-plane thermal conductivity and enhanced mechanical properties. Nanotechnology.

[24] Su K.-H., Su C.-Y., Chi P.-W., Chandan P., Cho C.-T., Chi W.-Y., Wu M.-K. (2021). Generation of Self-Assembled 3D Network in TPU by Insertion of Al_2_O_3_/*h*-BN Hybrid for Thermal Conductivity Enhancement. Materials.

[25] Jiang J., Zhou M., Li Y., Chen B., Tian F., Zhai W. (2022). Cell structure and hardness evolutions of TPU foamed sheets with high hardness via a temperature rising foaming process. J. Supercrit. Fluids.

[26] Kore S., Theodore M., Vaidya U. (2021). Effect of the Segmental Structure of Thermoplastic Polyurethane (Hardness) on the Interfacial Adhesion of Textile-Grade Carbon Fiber Composites. ACS Appl. Polym. Mater..

[27] Niu C., Luan C., Shen H., Song X., Fu J., Zhang L., Sun Y., Xu G., Ruan Z. (2022). Tunable soft–stiff hybridized fiber-reinforced thermoplastic composites using controllable multimaterial additive manufacturing technology. Addit. Manuf..

[28] Zhang X., Li T.-T., Wang Z., Peng H.-K., Lou C.-W., Lin J.-H. (2020). Facile fabrication and mass production of TPU/Silica/STF coated aramid fabric with excellent flexibility and quasi-static stab resistance for versatile protection. Prog. Org. Coatings.

[29] Lin M.-C., Lou C.-W., Lin J.-Y., Lin T.A., Lin J.-H. (2018). Mechanical property evaluations of flexible laminated composites reinforced by high-performance Kevlar filaments: Tensile strength, peel load, and static puncture resistance. Compos. Part B Eng..

[30] Zhang X., Li T.-T., Peng H.-K., Lou C.-W., Lin J.-H. (2021). Enhanced sandwich structure composite with shear thickening fluid and thermoplastic polyurethanes for High-performance stab resistance. Compos. Struct..

[31] Bates S.R., Farrow I.R., Trask R.S. (2018). Compressive behaviour of 3D printed thermoplastic polyurethane honeycombs with graded densities. Mater. Des..

[32] Rizzo F., Cuomo S., Pinto F., Pucillo G., Meo M. (2019). Thermoplastic polyurethane composites for railway applications: Experimental and numerical study of hybrid laminates with improved impact resistance. J. Thermoplast. Compos. Mater..

[33] El-Shekeil Y.A., Salit M.S., Abdan K., Zainudin E.S. (2011). Development of a new kenaf bast fiber-reinforced thermoplastic polyurethane composite. Bioresources.

[34] Suresha B. (2008). Friction and Dry Slide Wear of Short Glass Fiber Reinforced Thermoplastic Polyurethane Composites. J. Reinf. Plast. Compos..

[35] Hu J., Zhang Y., Liang C., Wang P., Hu D. (2021). The preparation and characteristics of high puncture resistant composites inspired by natural silk cocoon. Compos. Part A Appl. Sci. Manuf..

[36] Hu J., Liang C., Zhang Y., Li Y., Wang P. (2022). Preparation and characterization of flexible laminated composites impregnated with TPU/SiO_2_ for static puncture resistance. Int. J. Occup. Saf. Ergon..

